# Ocular Distribution and Pharmacokinetics of Lifitegrast in Pigmented Rabbits and Mass Balance in Beagle Dogs

**DOI:** 10.1089/jop.2017.0106

**Published:** 2018-11-14

**Authors:** Jou-Ku Chung, Elizabeth Spencer, Matthew Hunt, Thomas McCauley, Devin Welty

**Affiliations:** ^1^ Shire, Lexington, Massachusetts.; ^2^ Covance Laboratories, Inc., Madison, Wisconsin.

**Keywords:** dry eye disease, lifitegrast, mass balance, ocular distribution, pharmacokinetics

## Abstract

***Purpose:*** Lifitegrast is approved in the United States for the treatment of dry eye disease (DED). We assessed lifitegrast's ocular distribution/pharmacokinetic profile in rabbits, and ^14^C-lifitegrast mass balance/excretion in dogs.

***Methods:*** Female pigmented rabbits received a single topical ocular dose of lifitegrast (Formulation No. 1, *n* = 25; No. 2, *n* = 25) per eye twice daily (target, 1.75 mg/eye/dose). Blood/ocular tissues were collected on day 5. Beagle dogs received single intravenous (*n* = 10; target, 3 mg, 262 μCi/animal) and ocular (*n* = 8, target, 3 mg, 30 μCi/eye) doses of ^14^C-lifitegrast (∼8 weeks between doses). Blood, excreta, and cage rinse/wipes were collected. Concentrations were measured by mass spectrometry/liquid scintillation counting. Pharmacokinetic analyses (noncompartmental) included maximum concentration (*C*_max_), time to *C*_max_ (*t*_max_), and area under the concentration-time curve from 0 to 8 h (AUC_0–8_).

***Results:*** In rabbits, lifitegrast *C*_max_ and AUC_0–8_ were similar between formulations. *C*_max_ was highest in ocular anterior segment tissues: 5,190–14,200 ng/g [conjunctiva (palpebral/bulbar), cornea, anterior sclera]. Posterior segment tissues had lower concentrations (0–826 ng/g). AUC_0–8_ followed a similar trend. Plasma concentrations were low (*C*_max_ <18 ng/mL). Tissue/plasma *t*_max_ was ∼0.25–1 h. In dogs, after intravenous/ocular doses, ^14^C-lifitegrast was eliminated primarily through feces. Excreted radioactivity was mainly unchanged lifitegrast.

***Conclusions:*** High exposure of lifitegrast in rabbit ocular anterior segment tissues and low exposure in posterior segment tissues/plasma suggests that lifitegrast reaches target tissues for DED treatment, with low potential for off-target systemic/ocular effects. Excretion of unchanged ^14^C-lifitegrast suggests minimal drug metabolism *in vivo*. This is consistent with lifitegrast clinical trial efficacy/safety data.

## Introduction

Dry eye disease (DED) is an ocular disorder associated with surface tissue damage and impaired tear production and is commonly encountered in clinical practice.^1^ Although the etiology of DED is complex, there is strong evidence that supports chronic inflammation as a significant factor in the pathogenesis of DED.^1–4^ Several studies have shown that inflammatory mediators can be found in the ocular surface tissues of patients with DED, specifically in the corneal and conjunctival epithelium.^4–6^

Lifitegrast (Xiidra^®^; Shire, Lexington, MA) is a novel small molecule lymphocyte function-associated antigen 1 (LFA-1) antagonist that is approved by the U.S. Food and Drug Administration for the treatment of signs and symptoms of DED.^7^ Current understanding of the mechanism of action of lifitegrast is that it decreases T cell-mediated inflammation associated with DED by blocking the interaction between the integrin LFA-1 and intercellular adhesion molecule 1 (ICAM-1), thereby preventing T cell activation and recruitment.^8,9^ Lifitegrast is administered as a 5.0% ophthalmic solution (preservative free) applied to each eye twice daily (BID; ∼12 h apart).

The present report describes results from 2 separate studies: (1) lifitegrast's ocular distribution and pharmacokinetic (PK) profile in rabbits, and (2) a mass balance study of lifitegrast in dogs. The ocular distribution and PK study was carried out in female pigmented rabbits following repeated topical ocular dose administration of lifitegrast, and compared 2 dose formulations of lifitegrast used in phase 3 clinical trials (No. 1 in OPUS-1^10^ and No. 2 in OPUS-2^11^) to confirm that the slight differences in formulation did not result in significant differences in tissue distribution. Excretion and mass balance of lifitegrast were investigated following a single topical ocular or single intravenous dose of radiolabeled lifitegrast given to male and female beagle dogs. This work provides an understanding of the overall distribution profile of lifitegrast across the anterior and posterior ocular tissues and plasma, in addition to excretion profiles of lifitegrast after topical administration.

## Methods

### Study design

#### Rabbits

Female pigmented rabbits were assigned to receive 1 of 2 formulations of lifitegrast (Nos. 1 and 2) for 5 consecutive days. Each treatment group consisted of 25 rabbits, considered the minimum required to obtain scientifically valid results and ensure an adequate sample size for analysis. Animals received a single topical ocular dose of lifitegrast in each eye BID (days 1–4), ∼12 h apart (±1 h), at a target dose level of 1.75 mg/eye/dose (35 μL/eye/dose). On study day 5, animals were dosed in the morning only. Treatment groups 1 and 2 were dosed on separate days. The in-life portion of the study was conducted in May 2014.

#### Dogs

Beagle dogs received 2 doses of radiolabeled lifitegrast (1 intravenous and 1 topical ocular dose), with a 1-week washout period between doses. Target dose for each was lifitegrast 3 mg; the target dose volume differed for intravenous (10 mL) versus ocular (30 μL) administration, as did the target radioactive dose (262 μCi/animal and 30 μCi/eye for intravenous and ocular administrations, respectively). Following each administration, urine, feces, and cage rinse were assessed for radioactive content. The study was conducted August 15–November 1, 2007. This was part of a larger study that also included a third phase in which all tissues, including ocular tissues, blood, and plasma, were evaluated for distribution of radioactivity following topical ocular dose administration of radiolabeled lifitegrast (published previously).^12^

### Ethical conduct

Both the studies were reviewed and approved by the Institutional Animal Care and Use Committees at Covance Laboratories, Inc. to ensure that the studies were carried out in an ethical manner. The studies also adhered to the ARVO Statement for the Use of Animals in Ophthalmic and Vision Research and all procedures in the studies were conducted in accordance with the Animal Welfare Act Regulations (9 CFR 3). The dog study was conducted in accordance with the Food and Drug Administration Good Laboratory Practice Regulations (21 CFR 58) and the Wisconsin Department of Health and Family Services, Radiation Protection Section.

### Test animals

#### Rabbits

Fifty female New Zealand Red/White F1 Cross rabbits from Covance Research Products (Denver, PA) were used in the study. The animals were acclimated to study conditions for 13 days before dose administration. At dosing, the animals weighed 3,177–4,271 g and were ≥6 months of age. Animals were housed individually, in suspended stainless steel cages, with food and water *ad libitum*, and under a 12-h light/12-h darkness cycle throughout the study. Animals were not randomized, but were assigned to animal numbers based on overall health and the results of predose ophthalmic examinations. Ophthalmic examinations were performed by a board-certified veterinary ophthalmologist once at baseline (predose) using a Kowa handheld slit-lamp biomicroscope and an indirect ophthalmoscope with a condensing lens to ensure the study animals were healthy and had no relevant ocular findings pretreatment.

#### Dogs

Five male and 5 female purebred beagle dogs from Covance Research Products (Kalamazoo, MI) and 1 male purebred beagle dog from Covance Research Products (Cumberland, VA) were acclimated to study conditions for ∼2 weeks before dose administration. At dosing, the animals weighed 6.4–10.7 kg and were 6–7 months of age. Animals were individually housed in stainless steel metabolism cages with food and water *ad libitum,* and under a 12-h light/12-h darkness cycle. These cages enabled separation and collection of urine and feces. Animals were not randomized, but were assigned to study numbers based on body weight and overall health. Animals were checked for mortality and pain/distress in the morning and evening. General health and appearance were noted once daily. Baseline ophthalmic examinations were performed with dilation before the study began using a slit-lamp biomicroscope and an indirect ophthalmoscope. Animals were weighed at arrival, dose initiation, every 2 weeks through the study, and at study termination.

### Dose preparation and formulation

#### Rabbits

No formulation remained from either of the 2 phase 3 trials; formulations for the study that would be representative of the clinical material (confirmed by analytical data) were prepared at Covance Laboratories, Inc. In each formulation, lifitegrast was added to dose vehicle while stirring, and pH was adjusted with hydrochloric acid (HCl) and/or sodium hydroxide (NaOH). The formulation was stirred until a clear solution was obtained, filtered with a 0.22-μm filter (Millipore^®^ Millex-GV, 0.22 μm, Durapore^®^ PVDF; EMD Millipore, Billerica, MA) and stored at ∼5°C. Analysis of the formulations was performed by Almac Sciences (Souderton, PA), and concentrations were measured as 49.4 and 49.3 mg/mL for Formulation Nos. 1 and 2, respectively.

Formulation No. 1 (used in the OPUS-1^10^ trial): dose vehicle consisted of sterile water for injection, sodium chloride, sodium phosphate dibasic anhydrous, sodium bicarbonate, ethylenediaminetetraacetic acid (EDTA), and sodium thiosulfate pentahydrate, adjusted to a pH of 7.30 with HCl. After addition of lifitegrast, pH was adjusted to 6.90.

Formulation No. 2 (used in the OPUS-2^11^ trial): dose vehicle consisted of sterile water for injection, sodium chloride, sodium phosphate dibasic anhydrous, and sodium thiosulfate pentahydrate. After addition of lifitegrast, pH of the formulation was adjusted to 7.35.

#### Dogs

The intravenous dose included radiolabeled lifitegrast and sodium bicarbonate, which were combined on the day of dosing. Additional phosphate-buffered saline was added and the entire contents inverted to mix, then stirred magnetically for 70 min. The final pH of the solution was adjusted to 7.44 using dilute HCl and filtered through a 0.22-μm sterile filter.

The ocular dose included radiolabeled lifitegrast, nonradiolabeled lifitegrast, sodium bicarbonate, and preservative, which were combined on the day of dosing. The solution was inverted to mix and magnetically stirred for 2 min, followed by sonication for 70 min. Additional sodium bicarbonate was added to bring the admixture fully into solution. The final pH of the solution was adjusted to 6.83 using dilute NaOH and dilute HCl and filtered through a 0.22-μm sterile filter.

Duplicated weighed aliquots were taken from each dose formulation before and after dose administration. Aliquots were analyzed by liquid scintillation counting (LSC) to measure the radioactivity concentration and determine sample homogeneity.

### Dose administration

#### Rabbits

Topical ocular doses of lifitegrast (35 μL/eye/dose) were administered into the cul-de-sac of the eye via a calibrated positive displacement micropipette to ensure contact with the conjunctiva. The right eye was dosed first; collection times (below) were based on the time of dosing of the second (left) eye. Animals were not fasted before dose administration.

#### Dogs

The intravenous dose was calculated based on body weight measured on the day of dose administration. The volume of radiolabeled topical ocular dose was 30 μL/eye. The actual dose administered for both routes was calculated by weighing the syringe or pipette before and after administration. The intravenous dose was administered via a cephalic vein. The ocular dose was administered into the cul-de-sac of the eye via a micropipette, and eyelids were held together for ∼1 min. Each animal was restrained for 1–2 min to prevent rubbing of the eyes. Elizabethan collars were placed on each dog and remained on the animal through hour 2.

### Sample collection

#### Rabbits

Animals were euthanized with sodium pentobarbital, and blood and ocular tissues were collected from 5 animals per group per time point at 0.25, 0.5, 1, 3, and 8 h post last dose on study day 5. Blood (∼5 mL) was collected into tubes containing tripotassium EDTA (K_3_EDTA), an anticoagulant. Blood was centrifuged to obtain plasma. Aqueous humor and conjunctival tissues (bulbar and palpebral) were collected using fresh collection techniques. The following ocular tissues were collected using a frozen collection technique: choroid-retinal pigment epithelium (choroid-RPE), cornea, iris-ciliary body, lens, optic nerve, retina, sclera (anterior and posterior), and vitreous humor. Plasma and ocular tissues were stored at approximately −70°C.

#### Dogs

Blood (∼3 mL) was collected via a jugular vein into tubes containing K_3_EDTA from all animals predose and at 0.25, 0.5, 1, 1.5, 2, 4, 8, 24, 48, 72, 96, 120, 144, and 168 h postdose. Blood samples were placed in a chilled Kryorack (Streck Laboratories, Inc., Omaha, NE) or refrigerated until aliquoted and centrifuged to obtain plasma. Urine and feces were collected at hours 0–8 and 8–24 and at subsequent 24-h intervals until 168 h postdose. Urine and feces were collected in plastic containers stored at approximately −70°C. Cages were rinsed with water after each 24-h excreta collection through hour 144, and cage debris was collected. At 2 h postdose, each eye, the nasal area, and the Elizabethan collar also were wiped with gauze pads that were retained for radioanalysis (site wipes and collar wipes).

### Sample analysis

#### Rabbits

Lifitegrast concentrations were measured by a validated liquid chromatography tandem mass spectrometry (LC-MS/MS) method in rabbit plasma and multiple eye matrices (analyses were performed at ICON Development Solutions, LLC, Whitesboro, NY). The method was qualified for the analysis of rabbit aqueous and vitreous humor, using rabbit plasma as a proxy matrix. The LC-MS/MS analysis was performed using a Sciex API-5000™ mass spectrometer (SCIEX, Framingham, MA) coupled with a high-performance liquid chromatography (HPLC) system (Shimadzu Scientific Instruments, Somerset, NJ). The chromatographic separation was achieved on a Waters SymmetryShield™ RP18 HPLC column, 2.1 × 50 mm, 3.5 μm (Waters Corporation, Milford, MA), with a mobile phase gradient. The mass spectrometer was operated in turbo ionspray (positive ion) mode and the resolution setting used was unit for both quadrupoles Q1 and Q3. The lowest level of quantification for lifitegrast was 0.500 ng/tissue (the standard curve range was 0.5–100 ng/sample).

#### Dogs

Blood samples (duplicate weighed aliquots) were combusted and analyzed by LSC. The remaining blood was centrifuged at ∼2,400 rpm (1,300 *g*) for ∼10 min at ∼5°C, and the resulting plasma (duplicate weighed aliquots) analyzed by LSC. Urine and cage rinse samples were analyzed directly by LSC. A 50:50 mixture of ethanol and water was added to feces to facilitate homogenization. The sample was then combusted and analyzed by LSC. Sample combustions were done in a Model 307 Sample Oxidizer (PerkinElmer, Inc., Waltham, MA) and the resulting ^14^CO_2_ was trapped in a mixture of PermaFluor™ and Carbo-Sorb^®^. Sample radioactivity was measured in Model 2900TR liquid scintillation counters (PerkinElmer, Inc.) for ≥5 min or 100,000 counts. Each sample was homogenized before radioanalysis. All samples were analyzed in duplicate if sample size allowed. If results from sample duplicates (calculated as ^14^C dpm/g sample) differed by >10% from the mean value, the sample was rehomogenized and reanalyzed. Profiling and identification of metabolites versus unchanged lifitegrast and its metabolites was done by HPLC (Xterra MS C18 column; Waters Corporation) followed by liquid chromatography mass spectrometry (LC-MS) and LC-MS/MS (LTQ Orbitrap XL™ mass spectrometer with electrospray ionization [Thermo Fisher Scientific, Waltham, MA]; 610TR radiochemical detector [PerkinElmer, Inc.]).

### Pharmacokinetic analysis

Noncompartmental analysis^13^ was applied to the mean tissue and plasma lifitegrast concentration data. PK analyses included, wherever possible, determination of maximum concentration (*C*_max_) in ocular tissues and plasma, time to *C*_max_ (*t*_max_), area under the concentration-time curve (AUC) from time 0 to the last measurable time point (AUC_0–t_), and elimination phase half-life (*t*_1/2_). PK analysis was performed using Phoenix^®^ WinNonlin^®^ (Version 6.2; Pharsight Corporation, Sunnyvale, CA). Nominal doses and sampling times were used. Concentration values below the lower limit of quantification (BLQ; <0.500 ng/mL or <0.500 ng/sample, as appropriate) were treated as 0. If two-thirds of the samples were BLQ at a given time point, the mean was reported as “not calculated” in descriptive statistics and treated as 0 in the PK analysis.

## Results

### 

#### Rabbits

All animals (*n* = 50) had normal ophthalmic examinations predose. For animals receiving Formulation No. 1 (*n* = 25), mean (standard deviation [SD]) body weight was 3,710 (260) g and mean (SD) dose was 0.935 (0.0632) mg/kg. For animals receiving Formulation No. 2 (*n* = 25), mean (SD) body weight was 3,610 (242) g and mean (SD) dose was 0.959 (0.0658) mg/kg. Both formulations were well tolerated and no clinical safety observations were made during the study period.

Table 1 summarizes the PK parameters of lifitegrast for ocular tissues and plasma for each formulation. *C*_max_ and AUC from 0 to 8 h (AUC_0–8_) for the plasma and ocular tissues were generally similar between Formulation Nos. 1 and 2. For both formulations, lifitegrast exposure, as assessed by AUC_0–8_, was highest in the conjunctiva (palpebral). This was followed, in order of decreasing magnitude, by the cornea, sclera (anterior), conjunctiva (bulbar), sclera (posterior), iris-ciliary body, aqueous humor, and choroid-RPE. Concentrations of lifitegrast were highest in the anterior ocular surface tissues, with *C*_max_ for the conjunctiva (palpebral and bulbar) and cornea ranging from 5,930 to 14,200 ng/g for Formulation No. 1 and from 5,190 to 9,620 ng/g for Formulation No. 2. AUC_0–8_ for these tissues ranged from 13,400 to 30,800 ng·h/g and 12,000 to 36,600 ng·h/g, for Formulation Nos. 1 and 2, respectively. *C*_max_ of lifitegrast in the iris-ciliary body, aqueous humor, and choroid-RPE ranged from 79 to 190 ng/g for Formulation No. 1 and from 45.9 to 195 ng/g for Formulation No. 2. AUC_0–8_ in these tissues was 492–1,130 ng·h/g for Formulation No. 1 and 231–778 ng·h/g for Formulation No. 2.

**Table 1.** Pharmacokinetic Parameters of Lifitegrast Calculated for Various Ocular Tissues and Plasma for Each Formulation

	*Formulation No. 1*				*Formulation No. 2*			
*Tissue*	C_*max*_*(ng/mL or ng/g)*	t_*max*_*(h)*	*AUC_0-8_ (ng·h/mL or ng·h/g)*	t_*1/2*_*(h)*^a^	C_*max*_*(ng/mL or ng/g)*	t_*max*_*(h)*	*AUC_0-8_ (ng·h/mL or ng·h/g)*	t_*1/2*_*(h)*^a^
Conjunctiva (palpebral)	11,900	0.250	30,800	—	9,620	0.250	36,600	—
Cornea	5,930	0.250	25,500	—	5,190	1.00	15,200	—
Sclera (anterior)	11,200	0.250	17,500	1.97	5,870	0.500	11,200	2.32
Conjunctiva (bulbar)	14,200	0.250	13,400	2.02	9,370	0.250	12,000	—
Sclera (posterior)	826	0.250	2,360	—	369	0.500	1,570	—
Iris-ciliary body	190	0.250	1,130	—	195	1.00	778	—
Aqueous humor	79.0	3.00	530	—	89.5	1.00	340	—
Choroid-RPE	119	0.250	492	—	45.9	3.00	231	—
Plasma	17.4	0.250	11.2	0.850	9.52	0.250	16.4	—
Lens	3.85	1.00	5.44	—	0.794	3.00	NR	—
Optic nerve	36.0	1.00	NR	—	10.8	0.250	NR	—
Retina	31.2	1.00	NR	—	NR	NR	NR	—
Vitreous humor	2.09	0.250	NR	—	0.372	0.250	NR	—

^a^ Could not be calculated for most tissues due to the lack of a distinct elimination phase.

AUC_0–8_, area under the concentration-time curve from 0 to 8 h; choroid-RPE, choroid-retinal pigment epithelium; *C*_max_, maximum concentration; NR, not reported due to limited measurable data; *t*_max_, time to maximum concentration.

For the sclera, exposure to lifitegrast was significantly lower in the posterior tissue versus the anterior tissue. *C*_max_ of lifitegrast was 11,200 and 5,870 ng/g in the anterior sclera and 826 and 369 ng/g in the posterior sclera for Formulation Nos. 1 and 2, respectively. AUC_0–8_ of lifitegrast was 17,500 and 11,200 ng·h/g in the anterior sclera and 2,360 and 1,570 ng·h/g in the posterior sclera for Formulation Nos. 1 and 2, respectively. Limited measurable concentrations of lifitegrast were observed in the optic nerve, retina, and vitreous humor for both formulations. Mean *C*_max_ in these tissues ranged from BLQ to 36 ng/g, which was significantly lower than observed in anterior ocular tissues. AUC_0–8_ in the optic nerve, retina, and vitreous humor could not be calculated due to insufficient measurable data, which suggested that distribution to the back of the eye was very limited. Concentrations were also very low in the lens, with *C*_max_ = 3.85 ng/g and AUC_0–8_ = 5.44 ng·h/g for Formulation No. 1, and *C*_max_ = 0.794 ng/g (AUC_0–8_ not calculated) for Formulation No. 2. Across all tissues, *t*_max_ was generally between 0.25 and 1 h, indicating rapid absorption following topical ocular administration. Due to the lack of a distinct elimination phase, *t*_1/2_ in most ocular tissues could not be calculated, but in the conjunctiva (bulbar), *t*_1/2_ was 2.02 h (Formulation No. 1), and in the sclera (anterior), *t*_1/2_ was 1.97 and 2.32 h for Formulation Nos. 1 and 2, respectively.

**FIG. 1.**
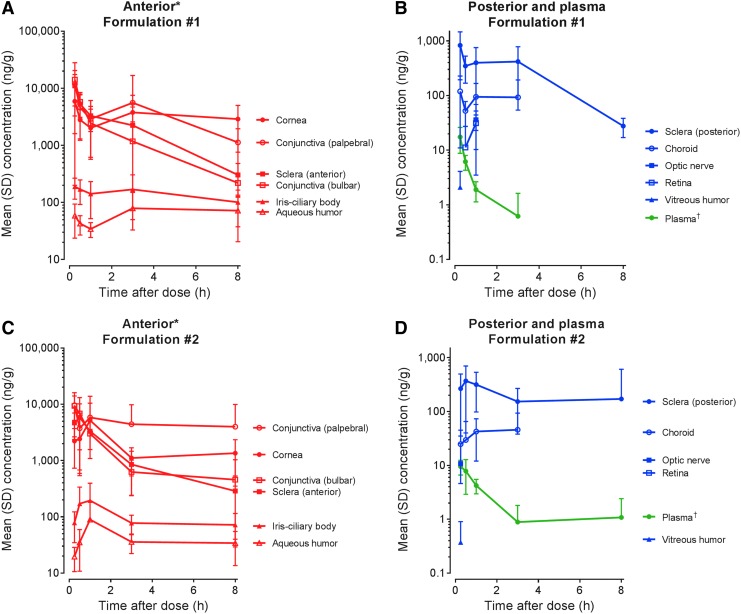
Mean (SD) concentration of lifitegrast in female pigmented rabbit ocular tissues and plasma at day 5. **(A)** Formulation No. 1: anterior segment tissues, **(B)** posterior segment tissues and plasma; **(C)** Formulation No. 2: anterior segment tissues, **(D)** posterior segment tissues and plasma. *Excludes lens, for which concentrations were <10 ng/g for both formulations. ^†^Concentration, ng/mL. SD, standard deviation.

Figure 1 shows mean concentrations of lifitegrast in the anterior (Fig. 1A, C; excluding lens) and posterior (Fig. 1B, D) ocular segment tissues over the 8 h postdose at day 5.

Low plasma levels of lifitegrast were observed (*C*_max_ values of 17.4 and 9.52 ng/mL, AUC_0–8_ values of 11.2 and 16.4 ng·h/g, for Formulation Nos. 1 and 2, respectively), following 9 doses (4 BID doses and 1 on day 5). After a topical ocular dose of Formulation No. 1, maximum plasma concentrations of lifitegrast were reached within 0.25 h (*t*_max_) and declined with a plasma *t*_1/2_ value of 0.850 h. Due to the lack of a distinct elimination phase, *t*_1/2_ in plasma for Formulation No. 2 could not be calculated (Fig. 1B, D).

#### Dogs

All animals were clinically healthy throughout the acclimation period and had normal ophthalmic examinations before dosing. The mean intravenous dose of radiolabeled lifitegrast was 2.96 mg/animal (262 μCi/animal), and the mean topical ocular dose was 1.75 mg/eye (30.0 μCi/eye). Due to a delay in dose preparation, the washout period between the intravenous and topical ocular doses was ∼8 weeks.

**FIG. 2.**
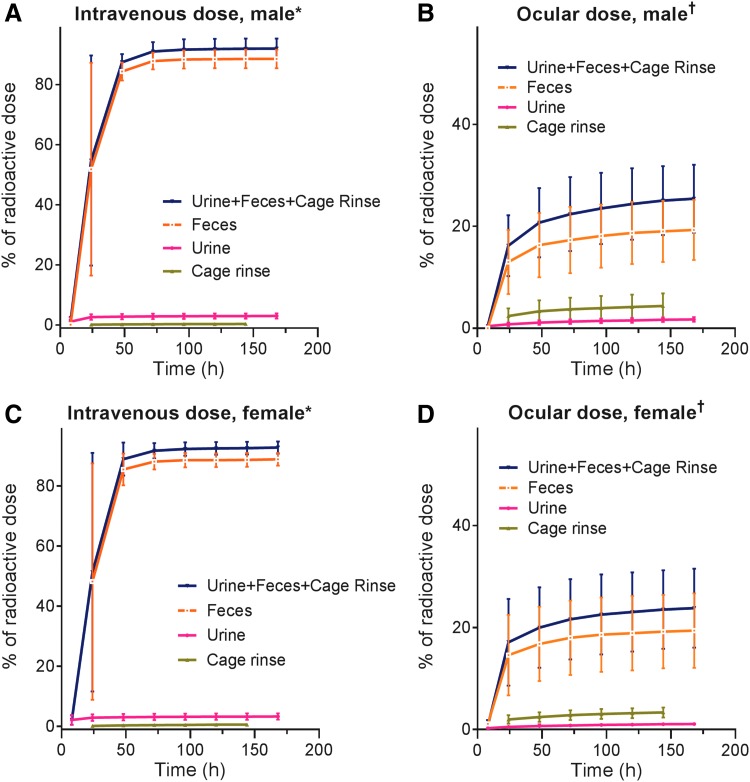
Mean (SD) cumulative percentage of radioactive dose recovered in urine, feces, and cage rinse from male **(A, B)** and female **(C, D)** beagle dogs after intravenous **(A, C)** or topical ocular administration **(B, D)** of ^14^C-lifitegrast 3 mg. *Male, *n* = 5; female, *n* = 5. ^†^Male, *n* = 4; female, *n* = 4.

Following intravenous administration, most of the radioactivity was excreted via feces (mean [SD] 88.6% [3.1%] in male dogs and 88.9% [2.1%] in female dogs). Urinary excretion accounted for a mean (SD) of 3.07% (0.86%) of the total administered dose in males and 3.31% (1.06%) of the total administered dose in females through 168 h (Fig. 2). Recovery of radioactivity in cage rinses, cage wash, and cage wipes accounted for <2.5% of the administered dose. For males and females, elimination was 90% complete within 48 h of dose. Total recovery of radioactivity through 168 h postdose ranged from 90.1% to 95.8% of the administered dose for males and from 91.1% to 95.7% of the administered dose for females. Figure 2A and C show the cumulative mean percentage excretion via urine, feces, and cage rinse following intravenous dosing for male and female dogs. Analysis by LC-MS and LC-MS/MS showed that the excreted radioactivity was mainly unchanged parent lifitegrast, accounting for ∼83%–85% of radioactivity in feces. Low levels of radioactivity (≤200 ng equivalents ^14^C-lifitegrast/g) were detected in the blood and plasma at the first time point (0.25 h postdose) for both males and females. Radioactivity declined rapidly within the first 8 h postdose and was approaching the lower limit of quantitation by 168 h postdose.

Following topical ocular administration, overall recovery of radioactivity was low, ranging from 20.4% to 38.1% at 168 h. Recovered radioactivity from cage rinses, wash, wipes, debris, site wipes (eye and nasal), and collar wipes was relatively high (up to ∼13% of the dose administered), suggesting that a large amount of the administered dose was lost through immediate loss due to spillage upon instillation, or through discharge from the nasal passages. As for intravenous administration, fecal elimination was the primary elimination route (mean [SD] 19.3% [5.9%] in males, 19.4% [7.3%] in females at 168 h), with most of the radioactivity recovered by 48 h postdose. Urinary excretion accounted for a mean (SD) 1.73% (0.44%) of the total administered dose in males and 1.08% (0.27%) of the total administered dose in females through 168 h. Figure 2B and D show the cumulative mean percentage excretion via urine, feces, and cage rinse following ocular administration in male and female dogs. Similar to intravenous administration, the majority of excreted radioactivity was unchanged lifitegrast. Blood and plasma levels of radioactivity were very low (<30 ng equivalents ^14^C-lifitegrast/g) at the first measurement time point (0.25 h postdose) and were BLQ by 8 h postdose.

## Discussion

**FIG. 3.**
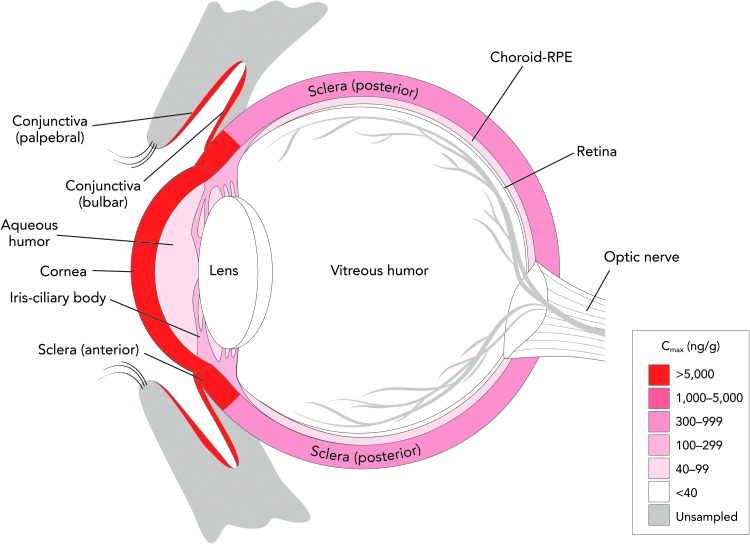
Lifitegrast ophthalmic solution 5.0% distributes with high concentration in the anterior ocular segment of rabbit eyes after repeated topical application. *C*_max_, maximum concentration.

Following repeated topical ocular BID dose administration of lifitegrast for 5 days in pigmented rabbits, distribution and exposure of lifitegrast was generally highest in the anterior ocular segment tissues, in particular the conjunctiva and cornea, while low concentrations of lifitegrast were observed in the posterior segment tissues (Fig. 3). Lifitegrast is approved for the treatment of DED, an ocular surface disorder in which T cell infiltration and inflammation have been observed in the conjunctiva and cornea.^4–6,14^ Thus, the predominant distribution of lifitegrast in these tissues corresponds well to the intended site of action, and is consistent with clinical effects on signs and symptoms of DED.^15,16^

Following single intravenous dose administration of radiolabeled lifitegrast in dogs, ∼90% of the radioactivity was eliminated in the first 48 h postdose, primarily through fecal excretion. Fecal excretion also was the primary route of elimination in dogs after a single ocular dose of radiolabeled lifitegrast, although the total recovery of the radioactivity was low. In addition, following both routes of administration, the excreted radioactivity was found to be mainly unchanged parent lifitegrast, suggesting minimal metabolism occurred *in vivo*. This finding was supported by an earlier *in vitro* study that showed that lifitegrast was metabolically stable when incubated with liver microsomes (data on file).

For animals receiving the topical ocular dose of lifitegrast, it is likely that a large amount of the administered radioactivity was lost through immediate spillage and discharge through the nasal passages. This conclusion is supported by the relatively high levels of radioactivity recovered in cage rinses and washes and the site wipes. Taken together, the ocular distribution in rabbits and excretion data in dogs suggest that topically administered lifitegrast distributes with high concentration in target ocular tissues for DED treatment while being rapidly eliminated from the body either through fecal excretion (unchanged) or discharge.

The distribution profile following repeat ocular BID administration in rabbits indicates low potential for off-target effects in the posterior ocular segment tissues. Plasma concentrations of lifitegrast also were notably low in rabbits and dogs, as observed previously in a phase 1 study in healthy volunteers^17^ and a subpopulation of the 1-year safety study SONATA,^18^ and plasma *t*_1/2_ in rabbits was short (0.850 h). Also, our data indicate that systemically absorbed drug is eliminated primarily by hepatic clearance as essentially unchanged drug and does not accumulate in any tissue. These data suggest limited potential for systemic side effects with lifitegrast. Consistent with these observations, the safety profile of lifitegrast in clinical trials has shown the drug to be generally well tolerated, with no suggestion of systemic toxicities.^10,11,16^ An additional point of note is that the ocular tissue elimination *t*_1/2_ determined in this study (eg, ∼2 h for the bulbar conjunctiva) supports the U.S. Food and Drug Administration-approved BID dosing of lifitegrast in humans.^7^ Because the dosing interval is long relative to the time needed to eliminate the drug from the ocular tissues, there is limited potential for drug accumulation. Distribution and exposure of lifitegrast in the plasma and ocular tissues were comparable between formulations, and both formulations were well tolerated with no clinically relevant safety observations in the study animals.

Lifitegrast has demonstrated efficacy for the treatment of signs and symptoms of DED across four 12-week randomized controlled clinical trials.^19^ However, in the phase 3 trials (OPUS-1 and OPUS-2) conducted with the 2 lifitegrast formulations under study, the efficacy outcomes in relation to signs and symptoms of DED were inconsistent.^15^ In the OPUS-1 trial (Formulation No. 1),^10^ there was a statistically significant treatment effect on the co-primary sign endpoint [inferior corneal staining score (ICSS)], but no statistically significant treatment effect on the co-primary symptom endpoint (visual-related function subscale of a symptom scale). In OPUS-2 (Formulation No. 2)^11^ on the other hand, there was a statistically significant treatment effect on the co-primary symptom endpoint (eye dryness score, visual analog scale), but no statistically significant effect on the co-primary sign endpoint (ICSS). It has been hypothesized that the different outcomes of the 2 trials might be due to different baseline DED severity levels for participants enrolled in these trials, and different disease etiologies among the trial participants.^15^ The similar ocular distribution and PK profiles of the 2 formulations evaluated in the rabbit study argue against the possibility that formulation of lifitegrast contributed to the different outcomes observed in the clinical trials.

The rabbit is the most common species used for evaluating ocular distribution because the rabbit eye is large enough to perform topical drug deliveries^20^ and comparable in size with a human eye. Pigmented rabbits of the crossed strain New Zealand Red/White F1 were used to increase comparability with humans by accounting for the potential for melanin to affect the distribution of the drug.^21^ Our study found relatively low lifitegrast concentrations in the iris-ciliary body relative to the conjunctiva and cornea, indicating that lifitegrast has relatively low potential for melanin binding. This is consistent with previous data on *in vivo* ocular penetration of ^14^C-lifitegrast in dogs that showed low lifitegrast concentrations in the iris^12^ and an earlier *in vitro* study that showed moderate binding of lifitegrast to melanin from *Sepia officinalis* (data on file). Further, we observed low lifitegrast concentrations in the choroid-RPE relative to the anterior ocular segment tissues, indicating that lifitegrast has low potential for binding to retinal pigments. This is consistent with previous dog study data that found retinal lifitegrast concentrations that were below the limit of quantification.^12^

Dogs, like rabbits, are commonly selected for ocular drug testing and evaluation.^20^ The size of their eyes and presence of melanin pigmentation makes them comparable with human eyes. Moreover, canine keratoconjunctivitis sicca may have the same pathogenic mechanism as DED, and DED drugs have been previously evaluated and tested in dogs.^12^

Previous investigational ocular PK studies of lifitegrast have been carried out in rats^22^ and dogs^12^ using radiolabeling. Consistent with our study, concentrations of radioactivity were found to be highest in the anterior segment tissues (bulbar conjunctiva, palpebral conjunctiva, and cornea) in dogs 30 min after topical administration.^12^ Similarly in rats, postdose radioactivity at 0.5 h was highest in the conjunctiva and cornea.^22^ Contrary to our findings, Rao et al.^22^ observed comparable radioactivity in the cornea versus the iris-ciliary body. A putative explanation is the difference in ocular anatomy between rodents and rabbits, including anterior chamber depth.^23^

A limitation of the ocular distribution study is that we did not evaluate tissue from the lacrimal gland, which also may be affected by inflammation in DED. Thus, it is not known whether lifitegrast distributes to and could potentially act in the lacrimal gland in addition to the ocular surface. Another limitation of this study was the lack of a distinct elimination phase, so *t*_1/2_ could not be calculated for most ocular tissues. Thus, the rate and relative extent of accumulation of lifitegrast in most ocular tissues could not be predicted. It should also be considered that no material from either of the phase 3 batch formulations remained, so fresh material (scaled down from the manufacturing process) was made as representative of the clinical material. In addition, interanimal variability could additionally have affected sampling and PK parameters. For the dog study, the excretion and mass balance data were limited by our inability to obtain good recovery of radioactivity after topical ocular dose administration.

## Conclusions

Following ocular administration in rabbits, the highest concentrations of lifitegrast are localized in anterior ocular segment tissues, in particular the conjunctiva and cornea, with low concentrations in the posterior segment tissues and plasma. The PK profile of lifitegrast was similar between formulations, indicating that the slight differences in formulations did not affect PK or tissue distribution of the drug. While we cannot be certain that the distribution of lifitegrast in humans will correlate with that in rabbits, the findings suggest that lifitegrast reaches the intended ocular surface tissues for DED treatment, while having limited potential for off-target systemic or ocular effects. The mass balance data in dogs suggest that the major route of elimination is through fecal excretion of unchanged parent lifitegrast. The results reported here are in agreement with clinical trial data that show that lifitegrast is effective in treating DED signs and symptoms, and is generally well tolerated with a favorable safety profile.
